# An Electrochromic Ag-Decorated WO_3−x_ Film with Adjustable Defect States for Electrochemical Surface-Enhanced Raman Spectroscopy

**DOI:** 10.3390/nano12101637

**Published:** 2022-05-11

**Authors:** Siqi Qu, Jing Guan, Dongqi Cai, Qianshuo Wang, Xiuyun Wang, Wei Song, Wei Ji

**Affiliations:** 1 School of Chemical Engineering, Dalian University of Technology, Dalian 116024, China; siqibinggan@mail.dlut.edu.cn (S.Q.); 21907028@mail.dlut.edu.cn (J.G.); 13942906490@mail.dlut.edu.cn (D.C.); 20201181003@mail.dlut.edu.cn (Q.W.); 2 State Key Laboratory of Supramolecular Structure and Materials, Jilin University, Changchun 130012, China; 3 College of Chemistry, Chemical Engineering and Resource Utilization, Northeast Forestry University, Harbin 145040, China

**Keywords:** SERS, defect states, EC-SERS, Ag-WO_3−x_ film

## Abstract

Electrochemical surface-enhanced Raman scattering (EC-SERS) spectroscopy is an ultrasensitive spectro-electrochemistry technique that provides mechanistic and dynamic information on electrochemical interfaces at the molecular level. However, the plasmon-mediated photocatalysis hinders the intrinsic electrochemical behavior of molecules at electrochemical interfaces. This work aimed to develop a facile method for constructing a reliable EC-SERS substrate that can be used to study the molecular dynamics at electrochemical interfaces. Herein, a novel Ag-WO_3−x_ electrochromic heterostructure was synthesized for EC-SERS. Especially, the use of electrochromic WO_3−x_ film suppresses the influence of hot-electrons-induced catalysis while offering a reliable SERS effect. Based on this finding, the real electrochemical behavior of p-aminothiophenol (PATP) on Ag nanoparticles (NPs) surface was revealed for the first time. We are confident that metal-semiconductor electrochromic heterostructures could be developed into reliable substrates for EC-SERS analysis. Furthermore, the results obtained in this work provide new insights not only into the chemical mechanism of SERS, but also into the hot-electron transfer mechanism in metal-semiconductor heterostructures.

## 1. Introduction

Electrochemical surface-enhanced Raman scattering (EC-SERS) spectroscopy is considered an ultrasensitive and non-destructive spectro-electrochemistry technique that provides mechanistic and dynamic information on electrochemical interfaces at the molecular level [1,2,3]. In EC-SERS, the Raman signal is mainly amplified by the enhanced electric field at a metallic surface owing to surface plasmon resonances, as well as chemical coupling between the molecule and the surface [4,5]. However, recent studies have found that plasmonic excitation can generate energetic hot electrons under specific conditions, thereby affording high catalytic activity for inducing chemical reactions at metallic surfaces [6]. Although this finding has pushed the development of plasmon-mediated chemical reactions in green photocatalysis, obtaining a reliable EC-SERS spectrum is difficult in actual measurements, which restricts detailed studies of molecular adsorption or chemical mechanisms at metal surfaces [7]. To avoid undesirable catalytic reactions, a low excitation power is recommended for EC-SERS measurements. However, it is generally difficult to obtain a high-quality spectrum in practical measurements because of the relatively weak Raman scattering signal under low laser power.

In comparison with metals, metal-semiconductor heterostructures have been demonstrated to exhibit superior SERS activity. The unique physical properties of semiconducting materials endow these composite SERS-active materials with multiple functions [8]. For example, ternary Ag-Cu_2_O/rGO nanocomposites serves as SERS substrates that enable both chemical sensing and monitoring of peroxidase-like catalysis [9]; whilst Ag-decorated TiO_2_ nanomaterials have been designed as recyclable, self-cleaning substrates for SERS detection of analytes [10,11]. As is well known, semiconducting materials have been widely used as supports to regulate the catalytic properties of metals [12,13]. The defect states in semiconducting materials can act as electron donors or electron acceptors. The metal-semiconductor heterostructures clearly provide a potential platform for constructing EC-SERS substrates for the mediation of plasmon-induced photocatalysis.

WO_3_ is a typical electrochromic cathode material, that can be reversibly switched between colorless and blue under positive and negative potentials, respectively [14]. The physicochemical mechanism behind the electrochromic behavior of WO_3−x_ films involves oxidation/reduction reactions induced by the electrochemical double intercalation/de-intercalation of cation (lithium or protons) and electrons into/out of the host WO_3_ lattice [15,16]. The dark blue color of the WO_3−x_ film is derived from the large amounts of reduced tungsten (W^4+^ and W^5+^) atoms in the lattice. In contrast with chemosynthetic WO_3−x_ samples, the concentration of defect states is generally much higher in electrochromic WO_3−x_ films, and, thus, new discrete, deep energy levels will be introduced into the bandgap during the electrochromic process. These deeper mid-gap states can act as recombination sites for trapping photogenerated electrons and holes [15,16]. Inspired by this possibility, we report the facile synthesis of a novel Ag-decorated WO_3−x_ (Ag-WO_3−x_) film, which exhibits both high electrochemical and SERS performance. More importantly, the Ag-WO_3−x_ film can inhibit plasmon-induced chemical transformation owing to the trapping of plasmon-excited hot electron in the deep energy levels of the WO_3−x_ film. Based on this finding, the electrochemical behavior of p-aminothiophenol (PATP) on Ag NPs surfaces is monitored for the first time.

## 2. Materials and Methods

### 2.1. Materials

Sodium tungstate dihydrate was obtained from Aladdin Chemistry Co. Ltd. (Shanghai, China). Oxalic acid dihydrate and potassium chloride were purchased from Tianjin Damao Chemical Reagent Factory (Tianjin, China). Other chemicals used in this work were acquired from Sinopharm Chemical Reagent Co. Ltd. (Shanghai, China). FTO glass was obtained from Wuhan Lattice Solar Energy Technology Co. Ltd. (Wuhan, China). All of the reagents were used as received without further purification.

### 2.2. Preparation of WO_3−x_ Film and Ag-WO_3−x_ Film

Na_2_WO_4_·2H_2_O (3.30 g) was dissolved in 50 mL deionized water, and the pH value of the solution was turned to 1.2 with HCl. Then, H_2_C_2_O_4_·2H_2_O (1.26 g) was added into the solution followed by addition of 50 mL deionized water. After stirring for 30 min, KCl (1 g) was added and aged for 24 h. This solution was used as a tungstic acid sol for growth of WO_3−x_ film on FTO glass. FTO substrates were cut into 2.5 cm × 3.0 cm and were ultrasonically cleaned with acetone, absolute ethanol, and deionized water for 30 min in turn. FTO substrates were activated in 0.1 M KOH solution with a 6 V voltage for 20 s. Subsequently, the above electro-activated FTO substrate was vertically immersed in tungstic acid sol for 10 min. The WO_3−x_ decorated FTO substrate was rinsed with deionized water and then dried with nitrogen gas. Finally, the as-prepared WO_3−x_ film was annealed in a muffle furnace at 300 °C for 1 h to obtain WO_3−x_ electrochromic film.

The prepared WO_3−x_ sample was first activated under different applied potentials (−0.1, −0.2, −0.3, −0.4, −0.5, and −0.6 V) in 0.1 M H_2_SO_4_ solution for 120 s. Subsequently, the activated WO_3−x_ sample was immediately immersed into the AgNO_3_ solution (0.01 g/mL) for 5 min. Finally, the Ag-WO_3−x_ sample was rinsed with deionized water and then dried with nitrogen gas.

### 2.3. Characterization of WO_3−x_ Film and Ag-WO_3−x_ Film

The field emission scanning electron microscopy (FE-SEM) images were collected with a HITACHI SU8020 field emission scanning electron microscope (Hitachi, Tokyo, Japan). The X-ray diffraction (XRD) pattern of sample was collected on a Rigaku SmartLab 9KW diffractometer (Rigaku, Tokyo, Japan) equipped with a Cu Kα radiation source. The UV-vis spectra were recorded on a PerkinElmer Lambda 1050+ UV-visible-near infrared light spectrophotometer (PerkinElmer, Waltham, MA, USA). The cyclic voltammetry (CV) and chronoamperometry (CA) were collected with a CHI660C electrochemical workstation (CH Instruments, Inc., Austin, TX, USA). The X-ray photoelectron spectroscopy (XPS) spectra were recorded on a Thermo Fisher Scientific THERMO X-ray photoelectron spectrometer (Waltham, MA, USA). The surface-enhanced raman spectroscopy (SERS) spectra were recorded on a Thermo Fisher Scientific DXR2 Confocal microRaman spectrometer.

## 3. Results

The Ag-WO_3−x_ film was synthesized on an FTO substrate through a two-step electrochemically activated procedure without the assistance of any additional reductant or surfactant (see details in the Section 2 Materials and Methods). As shown in Figure S1, the WO_3−x_ film was synthesized by following a previously reported procedure [17]. In this process, a blue amorphous tungsten trioxide hydrate film was formed on the FTO substrate via the reduction in polytungstic acid by the Sn layer obtained from the electrochemically activated FTO substrate (Figure S2). As shown in Figure S2e, only the XRD pattern from SnO_2_ was observed for the WO_3−x_ film sample, which can be ascribed to the FTO substrate. There is no obvious XRD pattern from WO_3−x_ film, which may indicate that the WO_3−x_ film represents an amorphous structure. After annealing at 300 °C for 1 h, the WO_3−x_ film was characterized by UV-vis-NIR absorption spectroscopy (Figure S3), cyclic voltammetry (Figure S4a), and chronoamperometry (Figure S4b). A detailed discussion of the characterization results is provided in the supporting information. The as-obtained WO_3−x_ film was further activated by applying a direct potential of −0.6 V for 120 s to generate reduced tungsten (W^4+^ and W^5+^), which provided the necessary reduction potential for decorating the film with Ag NPs [18]. When the WO_3−x_ film was dipped into an aqueous solution containing Ag ions, Ag NPs were grown on the WO_3−x_ films through the reaction of reduced tungsten with the Ag precursors at room temperature. This was accompanied by gradual oxidation of WO_3−x_ to WO_3_. The Ag-WO_3−x_ film was activated by applying a negative potential.

**Figure 1:**
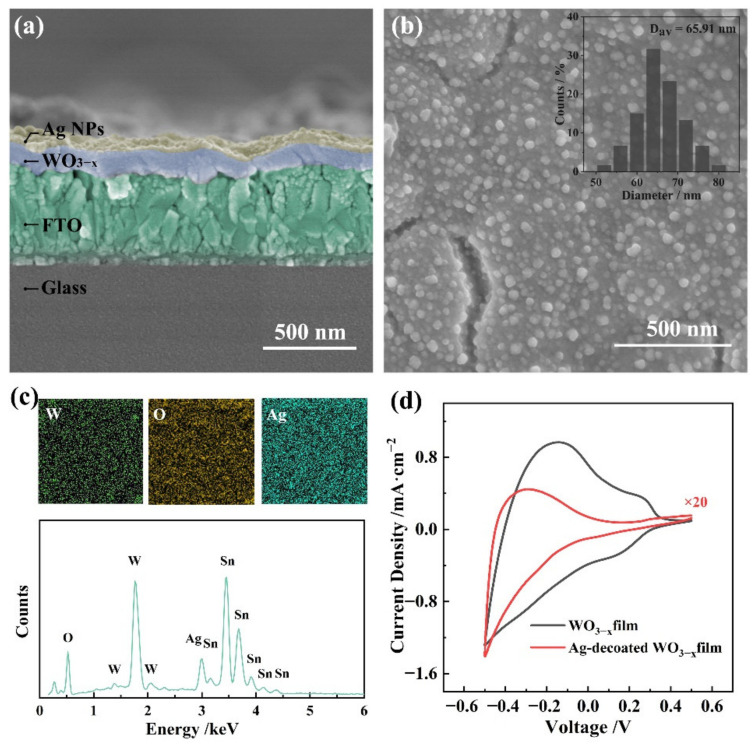
(**a**) Cross-sectional and (**b**) top view SEM images of Ag-WO_3−x_ film on FTO glass synthesized with an applied potential of −0.6 V. (**c**) Elemental mapping images and EDS profiles of Ag-WO_3−x_ film. (**d**) CV curves of WO_3−x_ film and Ag-WO_3−x_ film in 0.1 M H_2_SO_4_ at a scan rate of 50 mV·s^−1^.

Ag-WO_3_ films with different Ag NPs morphologies were prepared via in situ growth of the Ag NPs on the surface of the WO_3−x_ film by applying a series of activation potentials (−0.1 to −0.6 V) to the WO_3−x_ film. As shown in Figure 1 and Figure S5, increasing the activation potential increased the diameter and density of the Ag NPs on the WO_3_ film surface (Figure S6 and Table S1). Figure 1a shows a typical SEM image of the Ag-WO_3_ film under an activated potential of −0.6 V. The synthesized Ag NPs with a diameter of 65 nm were almost monodispersed on the WO_3−x_ film (Figure 1b), which are generally considered to be the best size for Ag-based SERS substrate. The energy-dispersive spectroscopy (EDS) profile of the substrate surface further confirmed that the newly formed particles were Ag NPs (Figure 1c). Figure S7 shows the UV-vis-NIR absorption difference spectra resulting from subtraction of the absorption spectrum of the WO_3_ film from that of the Ag-WO_3_ films synthesized by applying different activation potentials. Typical absorption associated with the Ag NPs was observed at approximately 490 nm. This wavelength of maximum absorption shifted to the longer wavelength side and became significantly more intense as the activation potential increased, corresponding to the increase in the size of the Ag NPs. These results demonstrate that the size and distribution of the Ag NPs are distinctly potential-dependent. This is attributed to the fact that the content of reduced tungsten is positively associated with the activation potential.

Cyclic voltammetry (CV) was used to investigate the electrochemical behavior of the Ag-WO_3−x_ film. Figure 1d shows the CV curves of both the WO_3−x_ film and Ag-WO_3−x_ film on the activated FTO substrate; the data were recorded in 0.1 M H_2_SO_4_ solution at a scan rate of 50 mV·s^−1^. The CV curve of the WO_3−x_ film shows a pair of reversible peaks located at +0.266/+0.135 V may be associated with the Sn^4+^/Sn^2+^ redox couple (+0.15 V vs. NHE) in the FTO film. The anodic peak at a potential of −0.143 V is related to the intercalation/de-intercalation of hydrogen ions, which is similar to previous reports for WO_3−x_ electrochromic films [17,18]. The anodic potential scan was followed by the double extraction of hydrogen ions and electrons, resulting in bleaching of the film due to the oxidation of W^5+^ to W^6+^. In contrast, the cathodic scan was followed by the reduction in W^6+^ ions and double injection of hydrogen ions and electrons, leading to coloration of the film. Decoration of Ag NPs on the WO_3−x_ film resulted in a negative shift of the anodic peak potential to −0.295 V. Moreover, the Ag-WO_3−x_ film showed a lower current density over the same time period compared to the WO_3−x_ film. These results indicate that the number of trap sites for cation intercalation/deintercalation in the WO_3−x_ film decreased significantly after decoration of the WO_3−x_ film surface with Ag NPs. However, the CV curve for the Ag-WO_3−x_ film is analogous to that of the WO_3−x_ film, indicating that the coloration/bleaching of the Ag-WO_3−x_ film is in accordance with the double intercalation/deintercalation of both hydrogen ions and electrons into/out of the WO_3−x_ films. Furthermore, no observe peak corresponding to the redox of Ag NPs was observed in the scanning range of −0.5 to +0.5 V. This may be because the WO_3−x_ film is easier to gain and lose electrons than that of Ag NPs, inhibiting the redox reaction of Ag in this scanning range.

**Figure 2:**
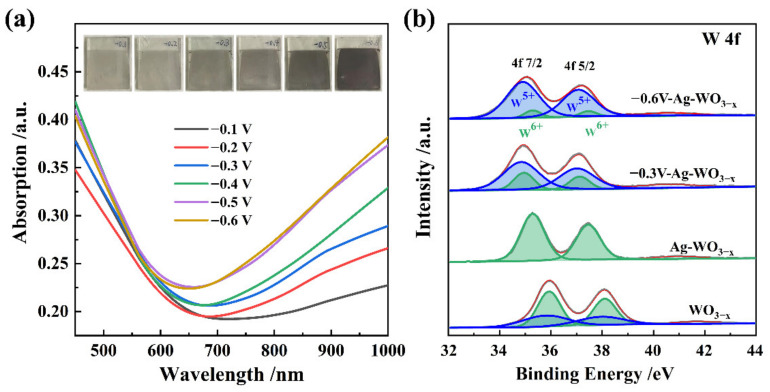
(**a**) Absorption difference spectra resulting from subtraction of the background absorbance from the absorption spectra of Ag-WO_3−x_ film acquired at different activated potentials. Inset shows the corresponding photographs of Ag-WO_3−x_ film. (**b**) XPS profiles of WO_3−x_ and Ag-WO_3−x_ at activation potentials of 0, −0.3, and −0.6 V.

The coloration/bleaching process of the Ag-WO_3−x_ film was further studied by UV-vis-NIR absorption spectroscopy (Figure 2a and Figure S8). Figure S8 shows the UV-vis-NIR spectrum of the Ag-WO_3−x_ film synthesized under different applied potentials. These spectra are the superposition of the absorption spectra of the Ag NPs and the WO_3−x_ film. Considering that the absorption spectrum of the Ag layer was not sensitive to the applied potential, the absorption spectrum of the Ag-WO_3−x_ film at an applied potential of 0 V was selected as the background absorbance. Figure 2a shows the absorption difference spectra resulting from subtraction of the background absorbance from absorption spectra of the Ag-WO_3−x_ film under an applied potential of −0.1, −0.2, −0.3, −0.4, −0.5, and −0.6 V, respectively. At different negative applied potentials, the Ag-WO_3−x_ films exhibited a broad absorption band in the NIR region. This absorption is associated with a reduction reaction, in which the oxidation state of WO_3_ changes from W^6+^ to W^5+^. As shown in Figure 2b and Figure S9, the synthesized WO_3−x_ film contained W^6+^ and W^5+^ ions. However, there was no obvious W^5+^ peak for Ag-WO_3−x_ after decoration with the Ag NPs, indicating that the Ag NPs were decorated on the WO_3−x_ film by oxidation of W^5+^ to W^6+^. The high-resolution XPS spectra of W4f can be deconvoluted into two pairs of peaks, which correspond to the binding energies of the W oxidation states, i.e., W^5+^ (located at 35.77 and 37.94 eV) and second doublet W^6+^ (located at 35.94 and 38.11 eV). The deconvoluted peak positions and intensities are given in Table S2. For the Ag-decorated WO_3−x_ film, the photoelectron peaks of W4f shift to lower binding energies, indicating that the strong electronic coupling interaction between Ag and WO_3−x_ film. Furthermore, the relative content of W^5+^ and W^6+^ changed in the activated Ag-WO_3−x_ films synthesized with different potentials (Table S2). Deconvolution of the XPS profiles confirmed that the content of W^5+^ increased as the applied potential became more negative. Accordingly, the XPS profiles further verified that the defect concentration in the Ag-WO_3−x_ film could be simply modulated by the applied potential.

The SERS performance of the synthesized Ag-WO_3−x_ films with different activation potentials was investigated using PATP as a probe molecule with 532 nm excitation (Figure S10, Supplementary Materials). No SERS signals were observed for the pure WO_3−x_ film, whereas SERS signals were observed after decoration of the WO_3−x_ film with the Ag NPs. The SERS spectrum of PATP on the Ag-WO_3−x_ film showed several strong peaks at 1140, 1384, and 1424 cm^−1^, which are characteristic Raman bands of *p,p′*-dimercaptoazobenzene (DMAB) [19,20,21]. This is attributed to the fact that the transformation of PATP to DMAB on the surface of the Ag NPs under visible light excitation occurs via a hot-electron-induced oxygen activation mechanism [22]. The SERS intensity increased with the activation potential. This behavior can be easily understood by acknowledging that the size and distribution of the Ag NPs increase with the activation potential. Considering the electrochemical stability of the WO_3−x_ film, the Ag-WO_3−x_ film synthesized with the activation potential of −0.6 V was selected for the following experiments.

**Figure 3:**
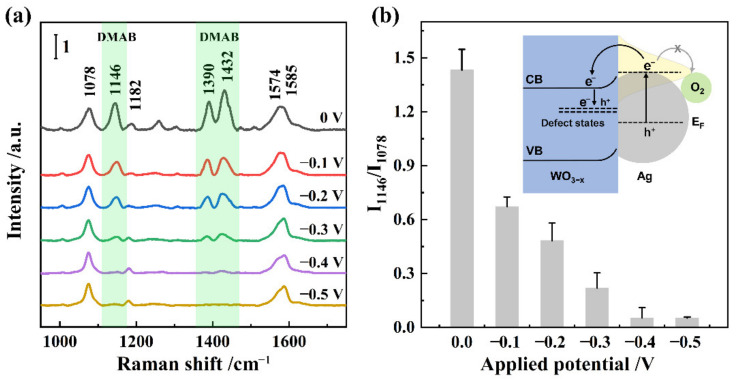
(**a**) SERS spectra of PATP on Ag-WO_3−x_ films synthesized with different activated potentials. (**b**) Band intensity ratio (I_1146_/I_1076_) as a function of activated potential. Inset shows the mechanism of hot electrons transfer in the Ag-WO_3−x_ system.

The Ag-WO_3−x_ films with different concentrations of defect states were obtained by re-activating the synthesized Ag-WO_3−x_ films under different applied potentials, after which the films were further modified with PATP molecules for the SERS measurements. Raman signals associated with DMAB were clearly observed for the Ag-WO_3−x_ film without activation (Figure 3a). This is because laser excitation of the plasmon resonance on the Ag NP surface could generate a hot electron-hole pair, followed by transfer of the hot electron into the adsorbed ^3^O_2_. Activated ^3^O_2_ triggers conversion of PATP to DMAB [21]. However, the Raman intensity of DMAB gradually decreased as the activated potential increased, and the Raman peaks ascribed to DMAB completely disappeared when the activation potential was more negative than −0.4 V, indicating that PATP cannot be converted to DMAB by oxygen. This result indicates that the photo-induced plasmonic hot electron is captured by the defect state of WO_3−x_, when the defect state is introduced into WO_3−x_ film by applying different potentials (Figure 3b). The band intensity ratio (I_1146_/I_1076_) was used to represent the relative amount of DMAB compared to that of PATP. The amount of DMAB was negatively correlated with the W^5+^/W^6+^ ratio, further confirming that plasmon-catalyzed oxidation on the Ag NPs can be inhibited by formation of the defect states in the WO_3−x_ film. This Ag-WO_3−x_ film provides a new EC-SERS platform for studying the adsorption behavior of PATP on the surface of Ag NPs.

## 4. Discussion

**Figure 4:**
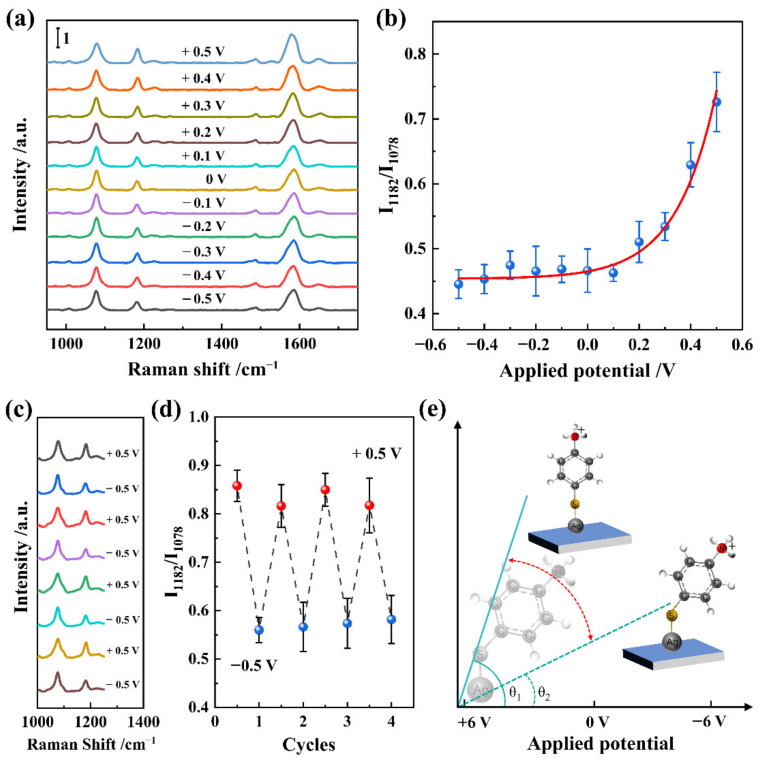
(**a**) In situ SERS profile of PATP adsorbed on Ag-WO_3−x_ film under different applied potentials. (**b**) Band intensity ratio (I_1182_/I_1081_) as the function of the applied potential. (**c**) Reproducibility of the potential-dependent SERS profiles under the applied potentials of +0.5 and −0.5 V. (**d**) Band intensity ratio (I_1182_/I_1081_) as the function of cycle number. (**e**) Physical model of PATP on the surface of Ag-decorated WO_3−x_ film under different applied potentials.

The applied potential-dependent adsorption of PATP on the surface of the Ag NPs surface was revealed by using the Ag-WO_3−x_ film (activated with a potential of −0.5 V) as an electrode. SERS measurements of the PATP-functionalized electrode were performed by scanning in the range of −0.5 to +0.5 V in phosphate buffer. Interestingly, the Raman bands ascribed to DMAB could not be observed for the Ag-WO_3−x_ film activated at a potential of −0.5 V, even when a positive potential of +0.5 V was applied (Figure 4a). This may be due to the fact that the activated defect state was embedded into WO_3−x_ and could not be fully removed by applying the positive potential. Notably, the Raman peak at 1182 cm^−1^, attributed to the C–H in-plane bending vibration, changed significantly with the applied potential, indicating a change in the adsorption behavior of PATP on the surface of the Ag NPs.

In accordance with the surface selection rules, vibrations normal to the substrate surface produce large signals, whereas vibrations parallel or tangential to the surface produce weak signals. Accordingly, for PATP molecules that were vertically adsorbed on the Ag-WO_3−x_ surface, the ring plane would be normal to the surface, and the corresponding in-plane vibration peak (such as the CH in-plane bending vibration at 1182 cm^−1^) would be enhanced. Because PATP molecules are adsorbed on the Ag-WO_3−x_ surface at a tilted angle, the peak of the in-plane vibration was less intense. In contrast, the CS stretching mode at 1078 cm^−1^ was less affected because the CS bond is close to the surface, resulting in relatively small changes in orientation. The relative intensity ratio of the CH in-plane bending versus the CS symmetric stretching vibration can be used as an index to evaluate the molecular orientation [23]. As shown in Figure 4b, there were no obvious changes in the relative intensity when a negative potential applied to the Ag-WO_3−x_ film. However, when a positive potential was applied to the Ag-WO_3−x_ film, the relative intensity increased gently from 0 to +0.5 V. To further verify the reversibility of the potential-dependent molecular orientation on the Ag-WO_3−x_ film, +0.5 and −0.5 V were alternately applied to the Ag-WO_3−x_ film (Figure 4c,d). The relative intensity was reversible, further confirming that the potential-dependent SERS is associated with the molecular orientation.

The in situ EC-SERS profiles were acquired in acidic solution, where the amine group of PATA was protonated and became positively charged. Under a positive potential, increasing the potential of the Ag-WO_3−x_ film increases the electrostatic repulsive force between the amine group and Ag-WO_3−x_ film surface, thus decreasing the tilt angle of the PATP molecules on the surface (Figure 4e). According to the surface selection rules, there is a relatively strong enhancement of the CH in-plane bending mode compared to the CS symmetric stretching mode. This expected result is identical to that observed in the EC-SERS profiles. Based on the above results, it is concluded that the potential-dependent SERS response of PATP molecules on the Ag-WO_3−x_ film is associated with the molecular orientation. Note that the potential-dependent SERS profiles observed in the present work are significantly different from those in previous works using Ag NPs surfaces, because the defect states in the WO_3−x_ film inhibit the hot-electron-induced oxidation of PATP during the EC-SERS measurements [24,25].

## 5. Conclusions

In summary, a novel Ag-WO_3−x_ electrochromic material was synthesized for EC-SERS. The electrochromic WO_3−x_ film suppresses the influence of a hot-electrons-induced catalysis, while offering a reliable SERS effect. This enabled detailed SERS investigations of the electrochemical behavior of molecules on metal NPs. This study should encourage the development of synthetic approaches for a EC-SERS substrate with dual functions. Furthermore, the results obtained in this work provide new insights not only into the chemical mechanism of SERS, but also into the hot-electron transfer mechanism in metal-semiconductor heterostructures.

## Data Availability

Not applicable.

## Supplementary Materials

The following supporting information can be downloaded at: https://www.mdpi.com/article/10.3390/nano12101637/s1, Figure S1: Schematic representation of the synthesis of Ag-WO_3−x_ film through a two-step electrochemical activation; Figure S2: SEM images of (a) FTO, (b) activated FTO, and WO_3−x_ film (c) before and (d) after annealing in air at 300 °C for 1 h. It can be seen that the FTO substrate has many diamond-shaped protrusions with a rough surface, and the protrusions become smooth after electrochemical activation (Figure S2a,b). A blue amorphous tungsten trioxide hydrate film was formed on the activated FTO substrate due to the reaction of Sn layer with the polytungstic acid (Figure S2c). After annealing, there is no other obvious change except some tiny cracks on the film surface (Figure S2d). The thickness of the WO_3−x_ film is estimated to be about 100 nm; Figure S3: UV-Vis-NIR absorption spectra and the corresponding photographs of the WO_3−x_ film under the applied potentials of −0.5V, −0.3V, −0.1V and 0.5 V. It can be seen that the degree of discoloration of WO_3−x_ film is deepened by the increase in negative potential, and the WO_3−x_ film returns to a colorless state when a positive potential is applied to the WO_3−x_ film. These results demonstrated the WO_3−x_ film has good electrochemical stability, and the content of reduced tungsten can be flexibly tuned by the applied potential; Figure S4: (a) CV curves of WO_3−x_ film in 0.1 M H_2_SO_4_ with a scan rate of 50 mV·s^−1^ for the 20 cycles. (b) Chronoamperometry curves of WO_3−x_ film by alternately applying a potential of ±0.5 V, 10 s for each state. The current density of the WO_3−x_ film only slightly decreases after 20 CV cycles, indicating that the WO_3−x_ film has good cyclic stabilities. Figure S4b compares the chronoamperometry curves of the WO_3−x_ film for the first three cycles and the last three cycles. There is no obvious change in the current response peak shape, which further confirmed that the WO_3−x_ film shows a good electrochemical stability; Figure S5: SEM images of the Ag-decorated WO_3−x_ film synthesized by applying of different activated potentials of (a) −0.1, (b) −0.2, (c) −0.3, (d) −0.4, (e)−0.5, and (f) −0.6 V; Figure S6. Size distributions of the Ag NPs on the synthesized Ag-WO_3−x_ film under the activation potential of (a) −0.1, (b) −0.2, (c) −0.3, (d) −0.4, (e) −0.5, and (f) −0.6 V; Figure S7: UV-Vis-NIR absorption difference spectra of Ag-decorated WO_3−x_ film synthesized under different applied potentials; Figure S8: The UV-Vis-NIR absorption spectra of Ag-decorated WO_3−x_ film under different applied potentials; Figure S9: (a) Full XPS spectra and (b) high-resolution XPS spectra of Ag3d for various of WO_3−x_ and Ag-WO_3−x_ samples; Figure S10: (a) SERS spectra of PATP adsorbed on Ag-decorated WO_3−x_ film synthesized under different activated potential. (b) Relative intensity of I_1146_/I_1080_ as a function of the activated potential; Table S1 The relationship between the activation potential and the diameter of synthesized Ag-WO_3−x_ film; Table S2: Fitting parameters (peak position, peak area and species percentage) for both W4f 7/2 and W4f 5/2 spectra of XPS taken from WO_3−x_ and Ag-WO_3−x_ activated with different applied potentials.
