# Supplementary material for: An Electrochromic Ag-Decorated WO3−x Film with Adjustable Defect States for Electrochemical Surface-Enhanced Raman Spectroscopy
Source: Nanomaterials (Basel). 2022 May 11;12(10):1637. doi: 10.3390/nano12101637 (PMC9146956; doi:10.3390/nano12101637)
Supplement: Supplementary file 1 [file nanomaterials-12-01637-s001.zip › nanomaterials-1694908-supplementary.pdf]

nanomaterials

Supporting Information

# An Electrochromic Ag-Decorated $WO_{3-x}$ Film with Adjustable Defect States for Electrochemical Surface-Enhanced Raman Spectroscopy

**Siqi Qu [1], Jing Guan [1], Dongqi Cai [1], Qianshuo Wang [1], Xiuyun Wang [1,*], Wei Song [2,*] and Wei Ji [1,3,*]**

[1] School of Chemical Engineering, Dalian University of Technology, Dalian 116024, China; siqibinggan@mail.dlut.edu.cn (S.Q.); 21907028@mail.dlut.edu.cn (J.G.); 13942906490@mail.dlut.edu.cn (D.C.); 20201181003@mail.dlut.edu.cn (Q.W.)
[2] State Key Laboratory of Supramolecular Structure and Materials, Jilin University, Changchun 130012, China
[3] College of Chemistry, Chemical Engineering and Resource Utilization, Northeast Forestry University, Harbin 145040, China
* Correspondence: xiuyun@dlut.edu.cn (X.W.); weisong@jlu.edu.cn (W.S.); jiwei@dlut.edu.cn (W.J.)

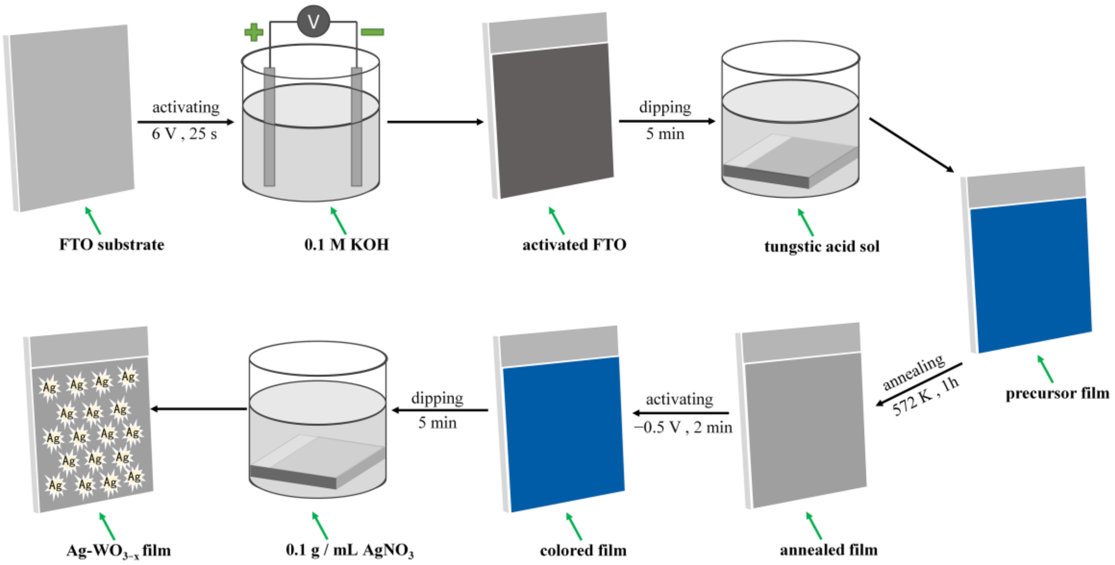


**Figure S1.** Schematic representation of the synthesis of Ag-$WO_{3-x}$ film through a two-step electrochemical activation.

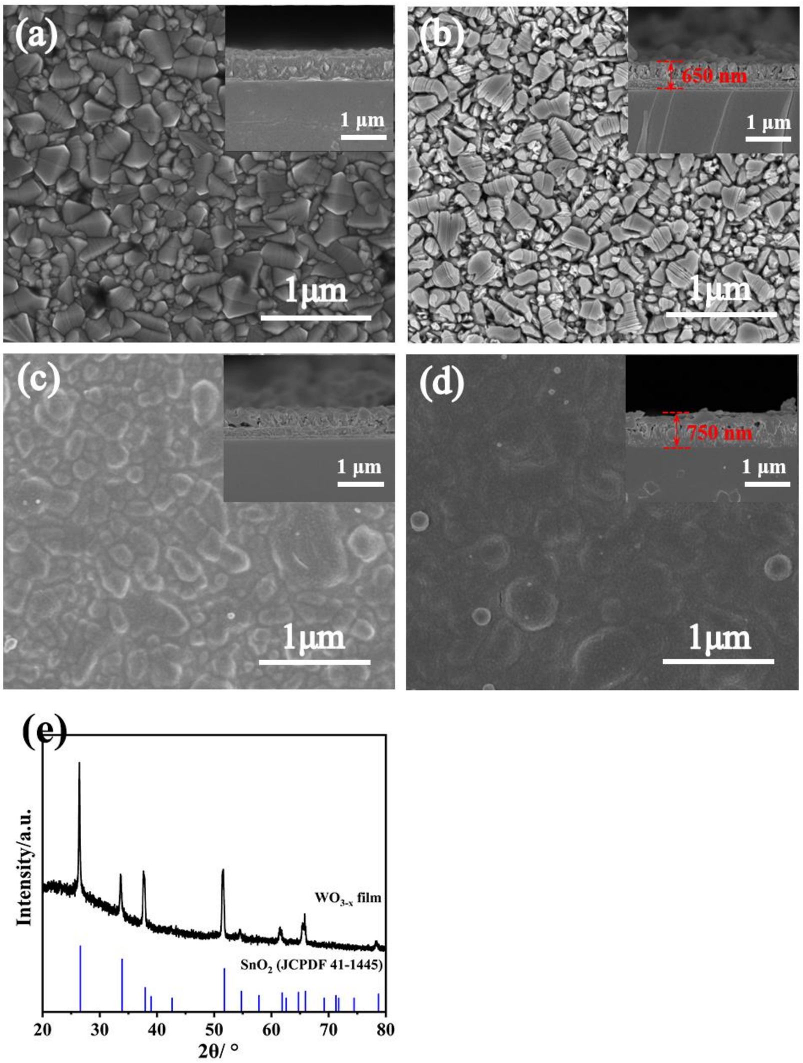


**Figure S2.** SEM images of (**a**) FTO, (**b**) activated FTO, and $WO_{3-x}$ film (**c**) before and (**d**) after annealing in air at 300 °C for 1 h. (**e**) X-ray diffraction (XRD) pattern of the synthesized $WO_{3-x}$ film. It can be seen that the FTO substrate has many diamond-shaped protrusions with a rough surface, and the protrusions become smooth after electrochemical activation (**a**,**b**). A blue amorphous tungsten trioxide hydrate film was formed on the activated FTO substrate due to the reaction of Sn layer with the polytungstic acid (**c**). After annealing, there is no other obvious change except some tiny cracks on the film surface (**d**). The thickness of the $WO_{3-x}$ film is estimated to be about 100 nm.

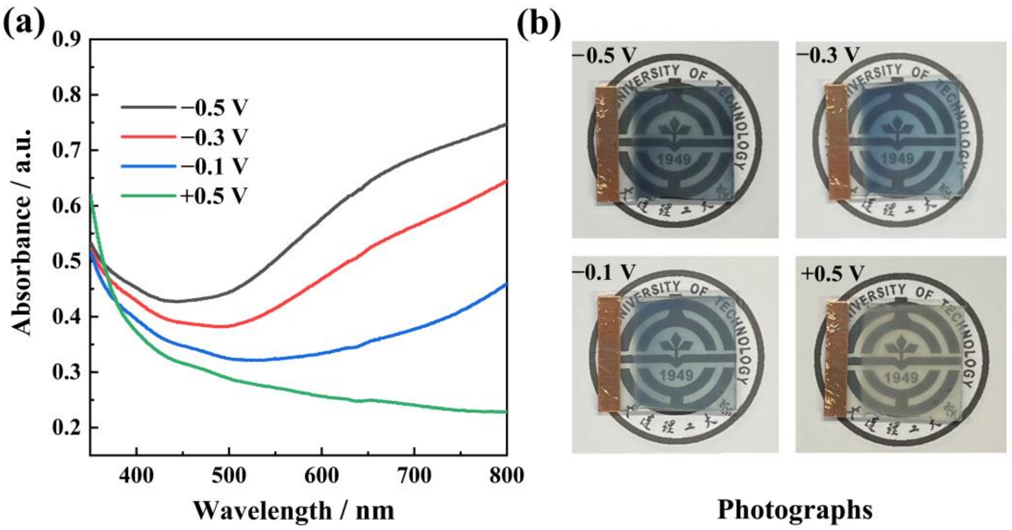


**Figure S3.** (**a**) UV-Vis-NIR absorption spectra and (**b**) the corresponding photographs of the $WO_{3-x}$ film under the applied potentials of −0.5V, −0.3V, −0.1V and 0.5 V. It can be seen that the degree of discoloration of $WO_{3-x}$ film is deepened by the increase of negative potential, and the $WO_{3-x}$ film returns to a colorless state when a positive potential is applied to the $WO_{3-x}$ film. These results demonstrated the $WO_{3-x}$ film has good electrochemical stability, and the content of reduced tungsten can be flexibly tuned by the applied potential.

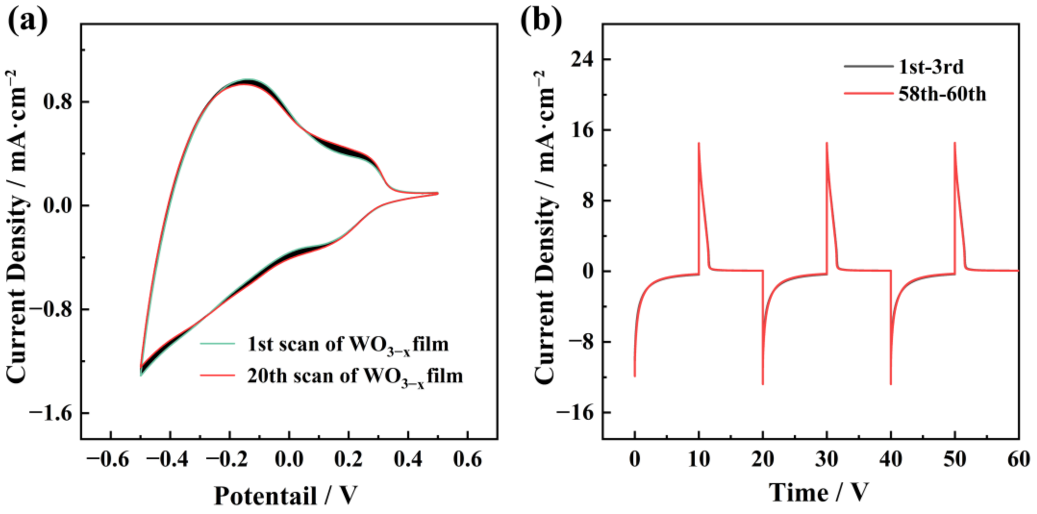


**Figure S4.** (**a**) CV curves of $WO_{3-x}$ film in 0.1 M $H_2SO_4$ with a scan rate of 50 $mV{\cdot}s^{-1}$ for the 20 cycles. (**b**) Chronoamperometry curves of $WO_{3-x}$ film by alternately applying a potential of ±0.5 V, 10 s for each state. The current density of the $WO_{3-x}$ film only slightly decreases after 20 CV cycles, indicating that the $WO_{3-x}$ film has good cyclic stabilities. (**b**) compares the chronoamperometry curves of the $WO_{3-x}$ film for the first three cycles and the last three cycles. There is no obvious change in the current response peak shape, which further confirmed that the $WO_{3-x}$ film shows a good electrochemical stability.

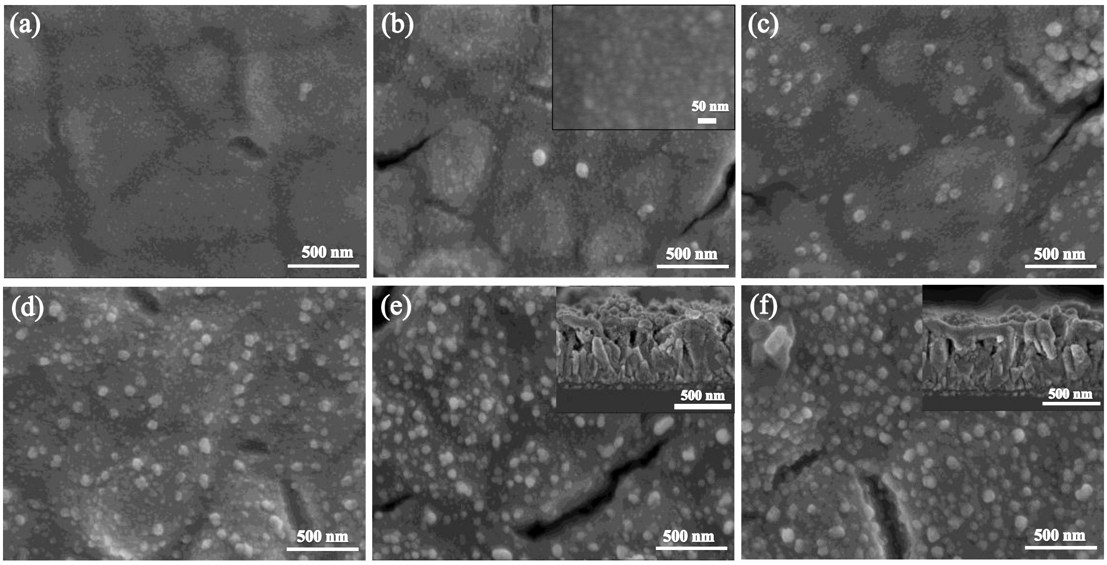


**Figure S5.** SEM images of the Ag-decorated $WO_{3-x}$ film synthesized by applying of different activated potentials of (**a**) −0.1, (**b**) −0.2, (**c**) −0.3, (**d**) −0.4, (**e**) −0.5, and (**f**) −0.6 V.

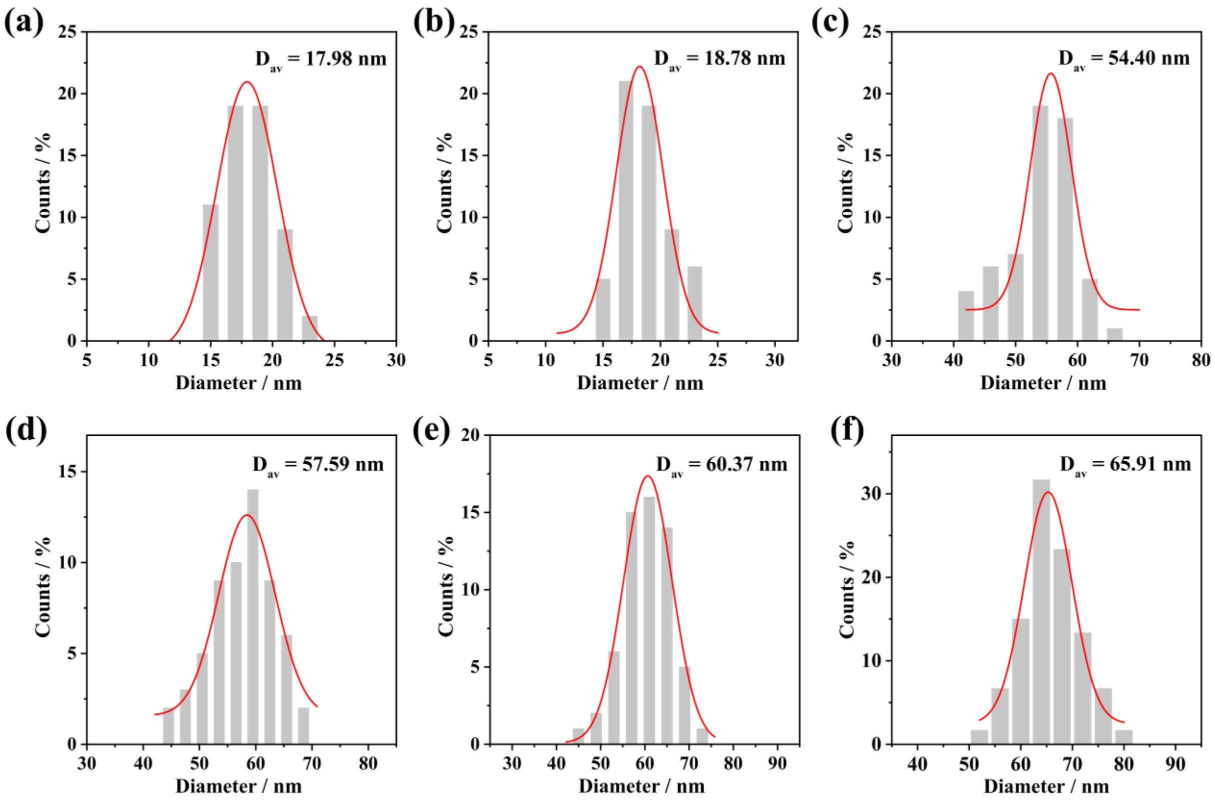


**Figure S6.** Size distributions of the Ag NPs on the synthesized Ag-$WO_{3-x}$ film under the activation potential of (**a**) −0.1, (**b**) −0.2, (**c**) −0.3, (**d**) −0.4, (**e**) −0.5, and (**f**) −0.6 V.

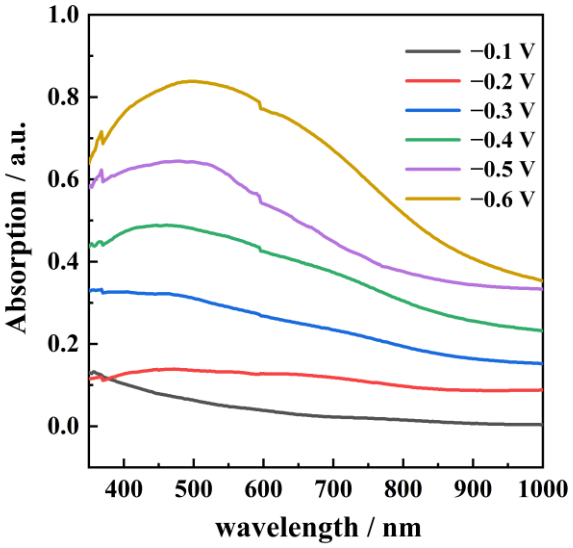


**Figure S7.** UV-Vis-NIR absorption difference spectra of Ag-decorated $WO_{3-x}$ film synthesized under different applied potentials.

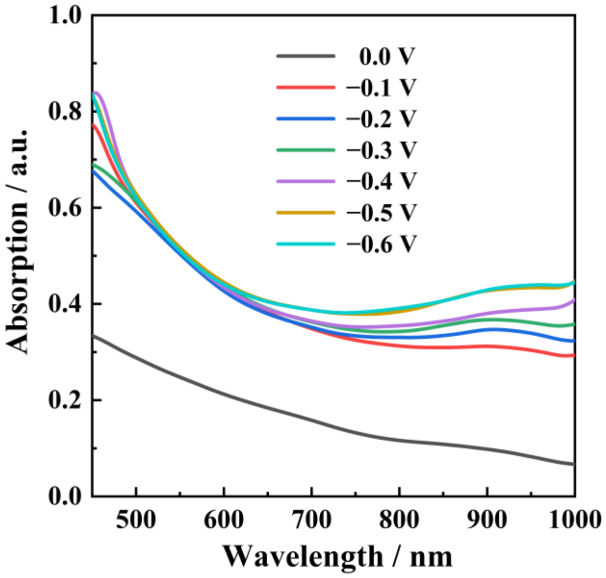


**Figure S8.** The UV-Vis-NIR absorption spectra of Ag-decorated $WO_{3-x}$ film under different applied potentials.

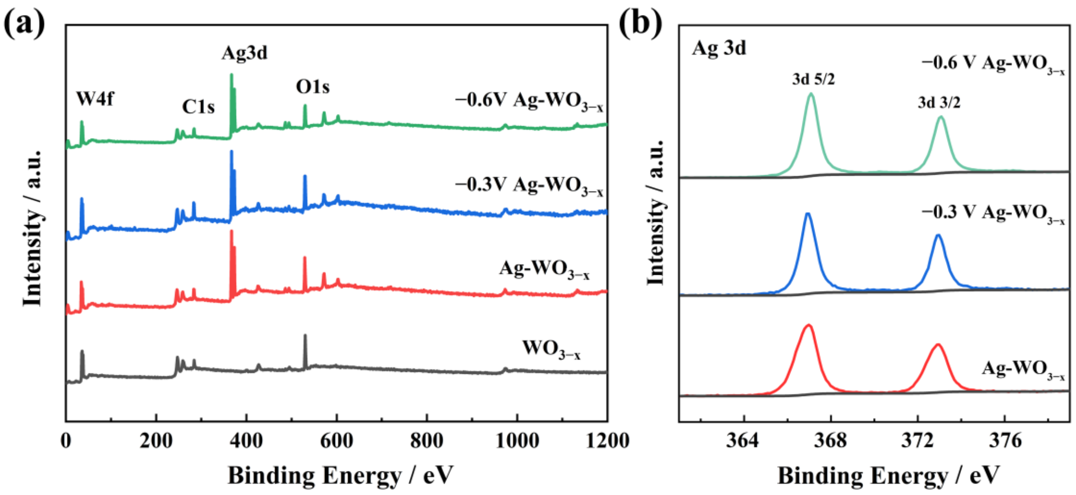


**Figure S9.** (**a**) Full XPS spectra and (**b**) high-resolution XPS spectra of Ag3d for various of $WO_{3-x}$ and Ag-$WO_{3-x}$ samples.

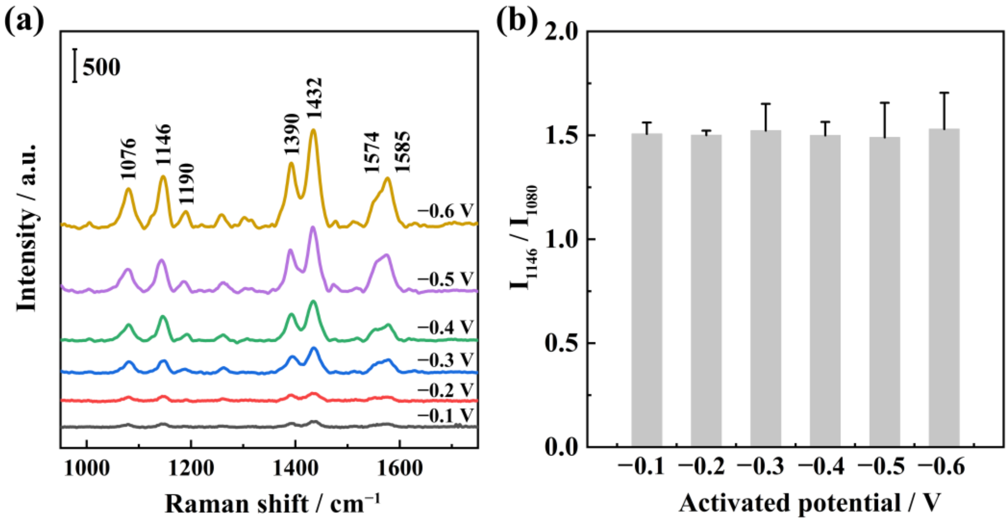


**Figure S10.** (**a**) SERS spectra of PATP adsorbed on Ag-decorated $WO_{3-x}$ film synthesized under different activated potential. (**b**) Relative intensity of $I_{1146}/I_{1080}$ as a function of the activated potential.

**Table S1.** The relationship between the activation potential and the diameter of synthesized Ag-$WO_{3-x}$ film.

| Activation Potential / V | −0.1 | −0.2 | −0.3 | −0.4 | −0.5 | −0.6 |
|---|---|---|---|---|---|---|
| Diameter of Ag NPs / nm | 17.98 | 18.78 | 54.40 | 57.59 | 60.37 | 65.91 |

**Table S2.** Fitting parameters (peak position, peak area and species percentage) for both W4f 7/2 and W4f 5/2 spectra of XPS taken from $WO_{3-x}$ and Ag-$WO_{3-x}$ activated with different applied potentials.

| Sample | Species | B.E. (eV) | | Area | | $W^{5+}/W^{6+}$ |
|---|---|---|---|---|---|---|
| | | 4f 7/2 | 4f 5/2 | 4f 7/2 | 4f 5/2 | |
| $WO_{3-x}$ | $W^{5+}$ | 35.77 | 37.94 | 17110.43 | 12832.82 | 0.61 |
| | $W^{6+}$ | 35.94 | 38.11 | 27981.36 | 20986.02 | |
| Ag-$WO_{3-x}$ | $W^{5+}$ | - | - | - | - | - |
| | $W^{6+}$ | 34.3 | 36.47 | 34332.16 | 25749.12 | |
| −0.3 V Ag-$WO_{3-x}$ | $W^{5+}$ | 34.85 | 37.02 | 18363.08 | 13772.31 | 3.18 |
| | $W^{6+}$ | 34.96 | 37.12 | 5781.302 | 4335.977 | |
| −0.6 V Ag-$WO_{3-x}$ | $W^{5+}$ | 34.9 | 37.07 | 64308.90 | 48231.67 | 11.30 |
| | $W^{6+}$ | 35.3 | 37.47 | 5692.382 | 4269.287 | |
